# Liver and Plasma Nesfatin-1 Responses to 6 Weeks of Treadmill Running With or Without Zizyphus Jujuba Liquid Extract in Female Rat

**DOI:** 10.5812/ijem.8438

**Published:** 2013-04-01

**Authors:** Abbass Ghanbari Niaki, Fatemeh Mohammadi Joojadeh, Navabeh Zare Kookandeh, Safar Najafi, Mohammad Javad Chaichi, Fatemeh Rodbari, Hasan Bayat

**Affiliations:** 1 Exercise Biochemistry Division, Department of Physical Education and Sport Sciences, University of Mazandarn, Baboulsar, IR Iran; 2 Department of Analytical Chemistry, Faculty of Chemistry, Mazandarn University, Baboulsar, IR Iran; 3 Department of cell and molecular biology, Faculty of Basic Sciences, University of Mazandran, Baboulsar, IR Iran; 4 Sina Laboratory, Mazandaran, Ghaemshahr, IR Iran

**Keywords:** Endurance Training, Nesfatin-1, Zizyphus Jujuba

## Abstract

**Background:**

Nesfatin-1 is a protein derived from a precursor molecule of the nucleobindin-2 gene, and acts as an anorexigenic peptide on food intake behavior, and its level isinfluenced by nutritional status, food composition [fat and carbohydrate (CHO)], and physical exercise.

**Objectives:**

The aim of this study was to investigate the effects of 6 weeks of treadmill running (at high intensity) program with and without zizyphus jujuba (high carbohydrate content) crud extraction on liver nesfatin-1, ATP, glycogen, and its plasma concentrations in female rats.

**Materials and Methods:**

Twenty-eight Wistar female rats (6-8 weeks old100-120 g of weight) were randomly assigned to saline-control (SC), saline-training (ST), zizyphus jujuba-control (ZJC), and zizyphus jujuba-training (ZJT) groups. Rats ran on a motor-driven treadmill at 35 m/min, 60 min/day, 5 days/week for 6 weeks. Animals received ZJ extraction and saline at the dose of 1.25 mL/100g of body weight. Seventy-two hours after the last training session rats were killed, a portion of liver excited, and plasma was collected for nesfatin-1, ATP, and glycogen measurements. A one-way ANOVA method, and Pearson correlation were employed. P < 0.05 was considered as significant.

**Results:**

A higher and significant liver nesfatin-1 level was found in ZJ groups (p < 0.005), but plasma nesfatin-1 responded differently. Changes in liver nesfatin-1 were accompanied with an increase in liver glycogen,but not ATP contents.

**Conclusions:**

The Findings indicate that higher liver nesfatin-1 and glycogen content by ZJ extraction might be due to the ZJ high CHO content, and it could be consideredas an anti-appetite herb.

## 1. Background

Nesfatin-1, a recently discovered protein derived from posttranslational processing of the nucleobindin 2 (NUCB2) gene is expressed in the appetite-control hypothalamic nuclei in rats (1). Nesfatin/NUCB2 is composed of a signal N-terminal peptide of 24 amino acids, and a protein structure containing 396 amino acids (2). It has been demonstrated that nesfatin-1 is expressed in the brain including the hypothalamic paraventricular nucleus (PVN), supraoptic nucleus (SON), arcuate nucleus, lateral hypothalamic area, and nucleus tractus solitarius in the brain stem which are involved in metabolic regulation, and feeding behavior (1-3). Nesfatin-1 is also expressed in extrahypothalamic tissues such as; rat gastric oxyntic mucosa or gastric X/A like cells (4, 5), digestive system (6), pancreatic beta cells (7), adipose tissue (8). In addition to serum/plasma, nesfatin-1 was detected in saliva and breast milk (9, 10). Nesfatin-1 has been suggested to act a novel inhibitor and potent regulator of food intake and body weight (4, 5), anti hyperglycemic, neuroendocrine regulator, and lowering body fat via appetite suppression (11-13). The expression of nesfatin-1, and its levels in serum/plasma have been shown to be affected by fasting and refeeding, restraint stress (14), abdominal surgery (4), and diabetes (15). In addition, the levels of plasma and tissues nesfatin-1 are also influenced by nutritional status, and food contents (high fat, high carbohydrate) (3, 8, 15-17). Recently, much more attention has been put on the alternative medicine, and using medicinal plant for appetite, body weight control, and also use as a strategy for this purpose (18-24). Ghanbari-Niaki et al., reported that the level of liver nesfatin-1 as an anorexigenic peptide was higher in magnolia-treated rats, and this elevated value was accompanied with higher liver glycogen concentration (25). On the other hand, the administration of pistacia atlantica (Baneh) crud extraction (with high essential oil content) reduced the nesfatin-1/nuclobindin-2 gene expression in rat small intestine (26). Zizyphus Jujuba (Chinese date/red date) is one of the most popular and well recognized herb in Chinese and Iranian traditional (27). Zizyphus jujuba (Rhamnaceae) is widely distributed in Iran, and fruit of this plant has gained wide attention in native herbal medicine for the treatment of a broad range of disorders. Chemical analysis of its fruit has shown the presence of flavonoids (quercetin and kaempferol), and phloretin derivatives (27-29). In addition to antioxidant contents, higher carbohydrate (glucose, fructose sucrose, and polysaccharide fractions) content was also determined in different species of zizyphus family, particularly Chinese and Iranian zizyphus jujuba by several investigators (30-32). Li et al. results showed that crude polysaccharides were composed of water (5.6%), protein (16.3%), ash (6.4%), fat (0.9%), starch (2.2%), fiber (15.7%), and soluble noncellulose polysaccharides (31.6%) (31). They also mentioned that analytical data of crude polysaccharides indicated soluble noncellulose polysaccharides extracted were most contaminated by co-extracted proteins. The constituting sugar analysis of crude polysaccharides revealed that arabinose (36.2 mol.%) was the predominant sugar residues, followed by mannose (22 mol.%), glucose (22 mol.%) and galactose (19.8 mol.%). Thus, considering the high carbohydrate content in zizyphus jujuba, and the effect of carbohydrate supplementation immediately after the exercise training on tissue energy source particularly, glycogen content, it is possible that the administration of zizyphus extraction might be effective on plasma and liver nesfatin-1 levels via an enhancement of liver glycogen concentrations.

## 2. Objectives

The current study was conducted to investigate the effects of 6 weeks treadmill running with our without oral administration of Zizyphus Jujuba liquid crud extraction on plasma and liver nesfatin-1 (as an anorexigenic peptide) response. The second purpose was to evaluate any possible change in nesfatin-1 with significant alterations in liver ATP and glycogen concentrations in female rats.

## 3. Materials and Methods

### 3.1. Plant Material

Suitable dried (at 25°C in dark place) zizyphus Jujuba fruits were provided by Dr Marzieh Saghebjoo our colleague in Birjand University from A Birjand medicinal herbs store . Then, the plant material was identified by the herbalist of herbarium collection of Biology department of Mazandaran University, Babolsar, Mazandaran, Iran. Seeds were removed, and 4 g of the seedless dried sample was finely chopped into small parts, and then extracted with 60 mL water for 70 h at 10°C, followed by filtration using a Whatman filter paper No.4. The filtered extract was centrifuged, and final volume of the extract was 40 mL (33). The extraction was prepared freshly twice a week. Zizyphus jujuba extract was administered orally to rats at a dose of 1.25mL/100g of body weight, immediately at the end of each training session, 5days/week, and for 4 weeks. Saline groups were also received the same volume of normal saline solution at the same situations.

### 3.2. Animals

All experiments involving the animals were conducted according to the policy of the Iranian convention for the protection of vertebrate animals used for experimental and other scientific purposes, and the protocol was approved by the Ethics Committee of the Sciences, University of Mazandaran (UMZ), and Babol University of Medical Sciences (BUMS, Mazandaran, Iran. Twenty eight Wistar female rats (6-8 weeks old100-120 g weight) were acquired from the Pasteur's Institute (Amol, Mazandaran), and maintained in the Central Animal House of the Faculty of Physical Education and Sports Science of UMZ. Seven rats were housed per cage (46-L) with a 12-hour,12-hour light-dark cycle. Temperature was maintained at 22°C ± 1.4°C. Diets (a pellet form) and water were provided *adlibitum*. Animals were randomly assigned into control (n = 14) and training (n = 14) groups. Rats were further divided into saline-control (SC), saline-training (ST), zizyphus jujube -control (ZJC), and zizyphus Jujube -training (ZJT). The control (SC and ZJC) groups remained sedentary; whereas the training groups underwent a moderate running exercise program (35 m/min (0% grade) for 60 min/day and 5 days/week for six weeks). The estrous cycle was determined in intact female rats by taking vaginal smears each morning by vaginal lavage. Smears were analyzed under a microscope to determine the type of cells present, and the stage of the estrous cycle. Only female rats showing at least two consecutive 4- or 5-day estrous cycles were used. The established estrous cycle in each female was used to select the day of the experiment, at which time the estrous cycle stage was confirmed by vaginal smear (34, 35).

### 3.3. Exercise Training Protocol

At first, the animals were familiarized with the rat treadmill apparatus, each day and for 4 days. The exercise groups were trained for 6 weeks on a motor driven treadmill as previously reported elsewhere (36, 37). The rats were submitted to run at 34 m/min for 60 minutes, 5 d/week. The animals were killed 72 hours after the last exercise session. Food but not water was removed from the cages 3hours before the killing. In our previous project we used male rats to investigate the effect of zizyphus jujuba extraction on nesfatin-1, and its gene expression in male rat different tissues. Thus we preferred to evaluate the effect of this extraction on female rats here. In this regard, we could provide some information for gender comparisons.

### 3.4. Tissue Biopsies

Seventy two hours after the last training session, rats were anesthetized with intraperitoneal administration of a mixture of ketamine (30– 50 mg / kg body weight), and xylazine (3– 5 mg / kg body weight). Liver tissue was excised, cleaned, divided into two pieces, washed in ice-cold saline, and was immediately frozen in liquid nitrogen, and stored at − 80 ° C. Blood was also collected in EDAT test tubes as anticoagulant, and immediately processed for plasma preparation, during a 10 min centrifugation at 3000 rpm. Plasma was stored at -80C too, for future analysis. To avoid the effect of the circadian rhythm, sampling began at 08.00AM, and completed at 11:30 AM.

### 3.5. Liver Glycogen, ATP and Liver, and Plasma Nesfatin-1 Concentrations

A large piece of liver was quickly excised using a freeze-champ tong previously cooled in liquid N2. The frozen liver was pulverized in a mortar to fine powder with frequent addition of N2. A portion (100mg) of powder was transferred to a plastic centrifuged tube containing 300µL of 30% KOH solution. The sample then digested by heating the tube for 20min in boiling water-bath, and following the digest is cooled and transferred to another test tube, and then diluted to the mark with water. The procedure was followed on the basis of Seifter et al. (38), and Carroll et al. (39) articles. Liver glycogen concentration was determined using an Anthrone reagent (38, 39). A Double Beam UV Spectrophotometer (Cecil Elegant Technology CE-5501 computing Cambridge England) was employed. A portion of frozen and powdered liver (50-75mg) was carefully homogenized, and extracted using a Phosphate-Buffered Saline (PBS, pH 7.4) solution (0.75-1.0mL) for liver Nesfatin-1 and ATP measurement (40, 41). Plasma and liver Nesfatin-1 were measured using a commercially available Rat Nesfatin-1 ELISA Kit (CUSABIO, Catalog No, CSB-E 1478r, China) sensitivity and Intraassay were 3.9pg/mL, and 7.5%, respectively. Liver ATP concentration was determined by a Bioluminescence method and using a commercial Brite TM ATP assay kit (BioVision Incorporated 155 S. Milpitas Boulevard, Milpitas, CA 95035 USA). The quantitation range was approximately between 1 nmol and 10 fmol/assay. A Sirius single tube luminometer (Sirius-e-05/06 Berthold detection systems GMBHPforzheim/Germany) was used.

### 3.6. Statistical Analysis

The Kolmogorov-Smirnov test was used to determine the normality of distribution, and variables were found to be normally distributed. All results were expressed as means ±SD. Statistical analyses were performed using a one-way analysis of variance. The least significant difference post hoc test was used in the event of a significant (P < .05) F ratio. Correlation was calculated using the Pearson Product Moment correlation. All statistical analysis was performed with SPSS (Version 16; SPSS, Chicago, IL).

## 4. Results

**Figure 1. fig2117:**
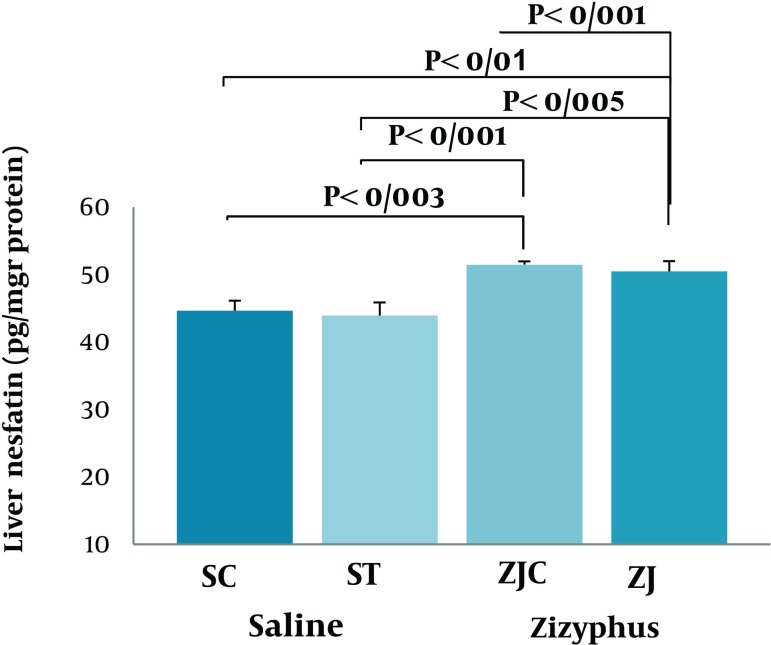
Liver Nesfatin-1 Concentrations in SC, ST, ZJC, and ZJT Groups of Wild-Type Female Rats. Data expressed as mean ± SD. Each column is for each group

Data analysis revealed significant differences in liver nesfatin-1 concentrations at the end of the treadmill running program (F = 6.938, P < 0.002). Using a proper post hoc test showed that liver nesfatin-1 concentrations were significantly (P < 0.003, and P < 0.005, respectively)higher in ZJC and ZJT groups, when compared to their counterparts ([Fig fig2117]Figure 1). A higher and significant nesfatin-1 concentration was observed in ZJ-treated animals when compared to S treated groups (P < 0.001) ([Fig fig2117]Figure 1).

**Figure 2.**
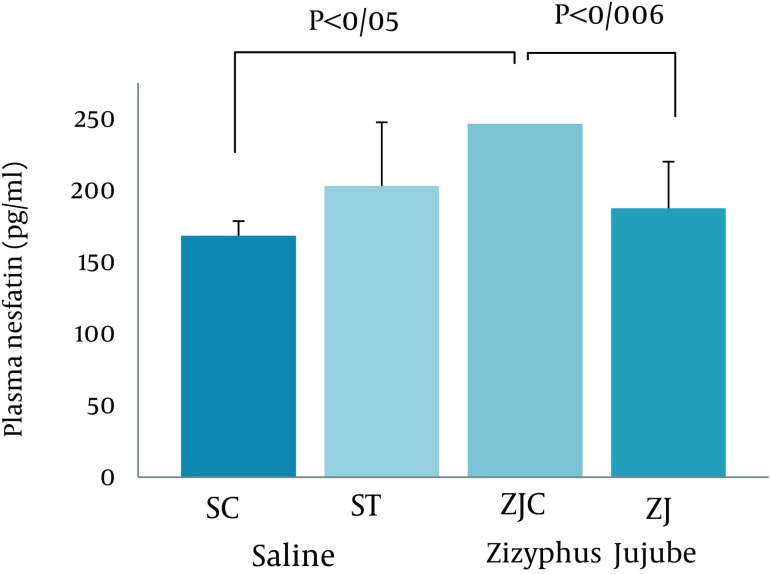
Plasma Nesfatin-1 Concentration in SC, ST, ZJC, and ZJT Groups of Wild-Type Female Rats.

Data analysis revealed significant difference in plasma nesfatin-1 concentration at the end of the treadmill running program (F = 5.853, P < 0.04). Higher and significant differences were observed between ZJC and ZJT, and SC (P < 0.006 and P = 0.05, respectively) (Figure 2).

**Figure 3.**
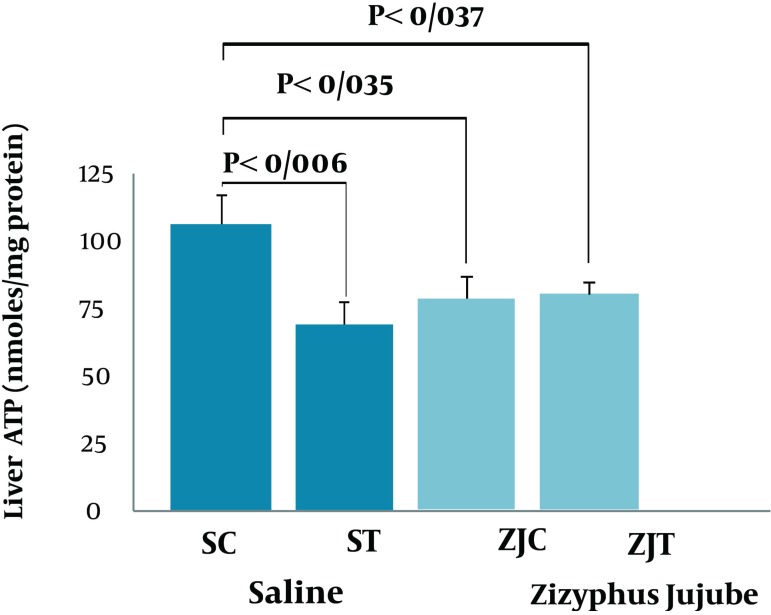
Liver ATP Concentration in SC, ST, ZJC and ZJT Groups of Wild-Type Female Rats. Data expressed as mean ± SD. Each column is for each group

**Figure 4.**
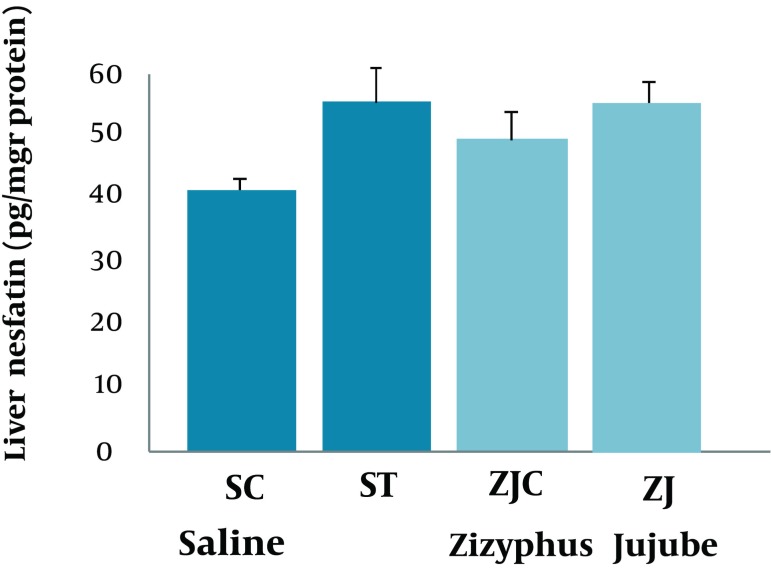
Liver Glycogen Concentration in SC, ST, ZJC, and ZJT Groups of Wild-Type Female Rats. Data expressed as mean ± SD. Each column is for each group

Changes in liver ATP concentrations were significant among groups (F = 3.33, P < 0.037) and a suitable following post hoc test indicated that SC group had a higher, and significant (P < 0.006, P < 0.035, P < 0.037, respectively) liver ATP concentration when compared to ST, ZJC, ZJT animals (Figure 3).There were no significant differences among the groups (F = 2.042, P < 0.30) (Figure 4). However, the levels of liver glycogen concentration were slightly higher in trained animals when compared to control groups. No significant correlations were found between the liver nesfatin-1 concentrations and plasma nesfatin-1 (*r* = -0.125; P < 0.267), liver ATP(r = -0.134; P < 0.252), and glycogen (r = 0.201; P < 0.158) concentrations.

## 5. Discussion

The main findings of the current study are as follows; 1) Higher liver nesfatine-1 concentrations have been found in both ZJC, and ZJT groups. 2) A higher plasma nesfatin-1 was observed in ZJC group when compared to SC group. 3) Changes in liver ATP concentrations were significant, and SC had higher liver ATP than other groups, but ZJ extraction restored ATP level in ZJT group to some extent. 4) Changes in liver nesfatin-1 were accompanied with higher liver glycogen content in ST, SJC, and SJT animals. 5) No significant correlations were found between the liver and plasma nesfatin-1 concentrations and measured variables. It has been reported that the injection of nesfatin-1 centrally, and peripherally inhibits food intake behavior in mice and rats (1-3, 42). Nesfatin-1 is also expressed in extrahypothalamic tissues such as; rat gastric oxyntic mucosa or gastric X/A like cells (4, 5), digestive system (6), pancreatic beta cells (7), adipose tissue (8). Osaki et al., reported that using a western blot method showed that nesfatin-1/nucleobindin-2 gene was expressed in the liver, pancreas, skeletal muscle (gastrocnemius ) subcutaneous, visceral fats, and intrascapular brown tissue in VMH, and sham operated rats (43). The levels of plasma and tissue nesfatin-1 are influenced by several factors such as; nutritional status (fasting and refeeding), diets composition (high carbohydrate, fat, and protein) (3, 15, 17, 44, 45), and different types of physical exercise (25, 46). Ghanbari-Niaki et al. observed a higher nesfatin-1/nucleobindin-2 gene expression in trained rat liver, hypothalamus, and soleus muscle tissues (26, 40). Rahmati-Ahmadabad et al. observed a higher and lower nesfatin-1/nucleobindin-2 gene expression in rats small intestine whose treated by saline, and pistacia atlantica (Baneh) extraction, respectively (26). Oh et al., reported that the concentration of nesfatin-1 was 60-80 ng/µg protein, and decreased up to 10-15 ng/µg protein in rat paraventricular nucleus of the hypothalamus (n = 4) in fed, and 24h after fasting period (1). Ramanjaneya et al. reportedthat the levels of nesfatin-1 secretion were around 90-110pg/mg total protein, and 25-35 pg/mg total protein in murine subcutaneous, and omental adipose tissues, respectively (8). There are very few articles focused on the effects of exercise training on tissues nesfatin-1 concentrations (25, 40, 41). Ghanbari-Niaki et al., reported a higher liver nesfain-1/nucleobidin-2 gene expression in trained male rat at the end of 8weeks of training program which was accompanied with a significant increase in liver content, but not liver nesfatin-1 concentration (0.2 ± 0.9 ng/g) (41). In other study by Ghanbari-Niaki et al., (2012) a change in rat hypothalamus nesfatin-1 concentration (4.68 ± 0.18 ng/g vs 4.77 ± 0.77 ng/g) was not significant in trained rats when compared to control animals (41). Ghanbari-Niaki reported that 6 weeks week treadmill running (25 m/min, 0% grade, for 60 min/day, 5 days/week) resulted a higher liver nesfatin-1 concentration (0.1-0.14 ng/g tissue) in trained rat liver treated with magnolia officinalis extraction when compared to saline treated animals. They also reported that a higher liver nesfatin-1 concentration was accompanied with a significant increase in liver glycogen, ATP, and TAC concentrations (25). The mechanisms by which endurance exercise training could change the nesfatin-1mRNA expression and its levels are not yet known. However, it has been suggested that nucleobindin-2mRNA, and nesfatin-1 protein expression in murine subcutaneous adipose tissue might be regulated by the tissue energy supply (8). It has also been suggested that fasting and feeding states decrease and increase nesfatin-1 concentration in rat PVN, and murine subcutaneous adipose tissues, respectively (1, 3, 8, 47). Fasting and refeeding have similar impacts on cellular energy status as exercise and carbohydrates, supplementation, especially on liver ATP and glycogen depletion, and replenishment (48-52). In the present study liver glycogen not ATP concentration was higher in trained rat treated with saline and Baneh solution. Although we did not measure plasma insulin, IL-6, and TNF-α concentrations, but according to Ramanjaneya et al., the levels of nesfatin-1 were increased ( from ~80 to 300 pg/mg of protein) after insulin, dexamethasone, (100nM), IL-6 (20ng/mL) administration, and decreased ( from~80 to 40pg/mg of protein) after the administration of TNF-α (10ng/mL) (8). Thus it seems that besides energy source, any change in the levels of some hormones and inflammatory markers might have impact on tissue and plasma nesfatin-1 concentrations. In summary, the results of the present study indicate that higher liver nesfatin-1 concentrations following the exercise training and ZJ supplementation were accompanied with more improvement in liver glycogen, but not liver ATP contents. In general, the findings showed that all measured variables in the ZJ-treated liver were higher than S groups. Considering the present results, and the nature of ZJ contents, we could also conclude that ZJ acts as a possible appetite suppressor via enhancing the liver and plasma nesfatin-1 levels in female rats. ZJ might also be considered as a candidate in liver glycogen super compensation induced by exercise training and ZJ supplementation. Our findings have provided some supports for the future studies on the current topic.
